# Nanoparticled Titanium Dioxide to Remediate Crude Oil Exposure. An In Vivo Approach in *Dicentrarchus labrax*

**DOI:** 10.3390/toxics10030111

**Published:** 2022-02-26

**Authors:** Patrizia Guidi, Margherita Bernardeschi, Vittoria Scarcelli, Paolo Lucchesi, Mara Palumbo, Ilaria Corsi, Giada Frenzilli

**Affiliations:** 1 Section of Applied Biology and Genetics and INSTM Local Unit, Department of Clinical and Experimental Medicine, University of Pisa, 56126 Pisa, Italy; margherita.bernardeschi@for.unipi.it (M.B.); vittoria.scarcelli@unipi.it (V.S.); paolo.lucchesi@unipi.it (P.L.); m.palumbo@studenti.unipi.it (M.P.); giada.frenzilli@unipi.it (G.F.); 2 Department of Physical, Earth and Environmental Science and INSTM Local Unit, University of Siena, 53100 Siena, Italy; ilaria.corsi@unisi.it

**Keywords:** crude oil, marine pollution, nanoparticles, nanoremedation, sea bass, *Dicentrarcus labrax*, DNA damage, comet assay

## Abstract

The contamination of marine water bodies with petroleum hydrocarbons represents a threat to ecosystems and human health. In addition to the surface slick of crude oil, the water-soluble fraction of petroleum is responsible for the induction of severe toxic effects at different cellular and molecular levels. Some petroleum-derived hydrocarbons are classified as carcinogenic and mutagenic contaminants; therefore, the oil spill into the marine environment can have long term consequences to the biota. Therefore, new tools able to remediate crude oil water accommodation fraction pollution in marine water are highly recommended. Nanomaterials were recently proposed in environmental remediation processes. In the present in vivo study, the efficacy of pure anatase titanium nanoparticles (n-TiO_2_) was tested on *Dicentrarchus labrax* exposed to the accommodated fraction of crude oil. It was found that n-TiO_2_ nano-powders themselves were harmless in terms of DNA primary damage, and the capability of pure anatase n-TiO_2_ to lower the levels of DNA damage induced by a mixture of genotoxic pollutant was revealed. These preliminary results on a laboratory scale are the prerequisite for deepening this new technology for the abatement of the cellular effects related with oil spill pollutants released in marine environments.

## 1. Introduction

Voluntary and accidental anthropogenic activities, synergistically with ongoing climate change, are causing an increase in disturbance of marine ecosystems. Eutrophication, overfishing, warming, ocean acidity and marine pollution represent multiple drivers operating at multiple spatial and temporal scales [1]. Marine pollution, especially oil spill-based, affects both marine and coastal environments with ecological and economic consequences. Accidents involving petroleum hydrocarbon spills frequently occur around the world, and even if the most striking feature of an oil spill in marine ecosystem is the surface slick of crude oil, the water-soluble fraction of petroleum is responsible for the induction of severe toxic effects at different cellular and molecular levels [2,3]. It was documented that some components of oxygenated oil spills, such as aromatic contents, acids and alcohols, can exert toxicity on a variety of targets, also causing chronic pollution of the aquatic environment [4].

Supporting this hypothesis, a biomonitoring study performed by Bolognesi et al. [5] highlighted that genetic damage is detectable in different indicator species present in the oil spill-impacted area many years after the accident responsible for the chemical release. This finding indicates that an enduring well of genotoxic compounds, possibly connected with chronic effects on the biota, potentially persists many years after the accident [5].

To mitigate the toxic effects induced by oil spill release, the application of nanotechnology has been presented as a suitable technique in remediating polluted matrices [6], although more attention has been paid to restoring soil rather than aquatic environments [7]. In fact, nanotechnology has already been used to improve water quality and to assist in environmental clean-up activities [6], relying on the fact that nanomaterials present enhanced reactivity due to their higher surface-to-volume ratio, and thus better effectiveness when compared to their bulkier counterparts.

Before employing nanomaterials in nanoremediation activities, it is important to assess their harmlessness with respect to the environment so that they do not act as pollutants themselves, posing an additional threat to the biota. For example, zero valent iron (nZVI) showed a great potential in the remediation of contaminated water, but it also exerted toxic effects during environmental applications [8].

In addition to nZVI, other nanomaterials such as nano-zinc, carbon nanotubes, and nano-titanium oxides have been proposed in environmental recovery techniques [9,10,11], but conflicting results have been reported concerning their potential toxicity [12]. Laboratory studies with in vivo models which aim to test the harmlessness of nanoparticles (NPs) employed in environmental remediation are necessary before any in situ application to recover polluted environments. Since titanium dioxide nanoparticles (n-TiO_2_) were described as able to adsorb several polycyclic aromatic hydrocarbons (PAHs) from water and soil [13], their potential to face water accommodated fraction (WAF) of crude oil toxicity in biological systems appears to be full of interest due to possible application in the restoration of oil spill-contaminated marine waters. A recent in vitro approach indicated that pure anatase nano-titanium was able to reduce genetic and chromosomal damage associated with environmental benzo(a)pyrene (B(a)P) exposure in marine mussel gill biopsies without exerting any cytotoxic and genotoxic effect [14]. However, the in vitro approach is represented by genotoxicity tests for screening substances and evaluating their initial safety, while the in vivo assay provides detailed information of biological and physiological significance [15]. The present preliminary in vivo study has been designed after obtaining in vitro results because the crystalline form of n-TiO_2_ pure anatase has never been used, to our knowledge, to assess its suitability for mitigating the genotoxic effects exerted by WAF crude oil in in vivo exposed fish.

As stated above, in order to assess the appropriateness of n-TiO_2_ in fighting the effects of WAF crude oil, in vivo specimens of juveniles of European sea bass, *Dicentrarchus labrax,* were exposed to n-TiO_2_ (pure anatase nano-titanium) alone and in combination with WAF crude oil. The novelty of this study relies on the application of pure anatase nano-titanium to study its potential ability to counteract the genetic damage induced by WAF of crude oil using an in vivo approach. The usefulness of the in vivo genotoxicity results relies on the fact that they can reflect absorption, excretion, distribution and metabolism of chemicals, while the in vitro test limits itself to cellular effect investigation [16].

The potential genotoxic effects exerted by the different substances utilized was investigated through the comet assay. It is a well-known test able to detect primary repairable, and therefore reversible DNA damage; in this context, it was applied in its mild alkaline version in order to detect exclusively single strand breaks on *D. labrax* erythrocytes [17]. The comet assay is a technique based on single cell analysis. The use of peripheral erythrocytes avoids the complex procedures associated with tissue dissociation and animal sacrifice. In addition, high mitotic rate of hematopoietic tissues provides a rapid response to genotoxic exposure, making fish erythrocytes a suitable cell model for genotoxicity [18] tests in the nano-geno-toxicology research field.

## 2. Materials and Methods

### 2.1. Chemicals

Low melting agarose (LMA), normal melting agarose (NMA), ethidium bromide (EtBr), Triton X-100, ethylenediaminetetraacetic acid (EDTA), Trizma base, dimethyl sulfoxide (DMSO), NaOH, Tris-HCl, and trypan blue were purchased from Sigma-Aldrich, (Darmstadt, Germany). Pure anatase nanosized TiO_2_ (25 nm), (n-TiO_2_) was kindly supplied by Eigenmann & Veronelli (Milan, Italy) CAS NUMBER: 1317-70-0. A bath-type sonicator, at 35 kHz (Transonic 460/H, Elma, Singen, Germany), was used for sonication. Jeol 100 SX (Milan, Italy) was used for transmission electron microscope (TEM) analysis.

### 2.2. Preparation and Characterization of NPs

Pure anatase TiO_2_ nanosized (declared purity of 99.9%) characterization data were reported in a previous study [14]. n-TiO_2_ stock suspension was prepared in filtered (0.22 µm) artificial sea water (ASW) (10 mg/mL) with 32% salinity and pH 8 ± 0.1, sonicated for 30 min into reduced aggregation, and serially diluted immediately before exposure (100 W, 50% on/off cycle). Immediately after that, stock suspension was diluted in ASW to obtain the working suspension, which was again sonicated for 15 min prior to use as previously reported with regard to another n-TiO_2_ crystalline form investigated by our group [19]. Even though the types of n-TiO_2_ investigated are different, the experimental procedure to avoid particle aggregation was the same. In order to assess the effectiveness of the sonication procedure, stock solution was diluted 1:10 and dropped onto a 150-mesh formvar carbon-coated nickel grid, air dried and observed with TEM.

### 2.3. In Vivo Exposure

The water accommodated fraction (WAF) of crude oil was prepared according to the work of Rial et al. [20], with modified concentrations used by Kerambrum et al. [21]. Briefly, 1.7 g crude oil were dissolved in 250 mL of ASW in a 250 mL Duran with Teflon cap (loading rate 1.36 g/20 L). The mixture was kept on a magnetic stirrer for 24 h and in the dark at 20 °C. A double volume of WAF was prepared, and thereafter 200 mL of the solution were mixed with 20 L ASW to obtain the treatment tank (both crude oil alone and in co-exposure with n-TiO_2_). The experiment was run in artificial sea water (ASW) and prepared following International Standard Guide [22] in order to test both WAF crude oil and n-TiO_2_ genotoxic potential and nanoparticles remediation capacity in high ionic strength media, avoiding any potential additional contamination generally occurring in natural sea water. Taking into consideration international guidelines concerning in vivo genetic toxicology testing using comet assay, where a minimum of four scorable animals are recommended in each dose group at each sample time [23,24] and after an acclimatization period of 24 h in artificial sea water (ASW), 20 juveniles of European sea bass *D. labrax* (Linnaeus, 1758), 5 specimens for each experimental group, with comparable dimensions (LT 10 ± 1 cm) were split in 4 independent tanks (20 L each one): control (ASW), crude oil (1.36 g/20 L), n-TiO_2_ (10 mg/L) and in combination (crude oil + n-TiO_2_, co-exposure). To treat the co-exposure tank, nano-TiO_2_ was added to the WAF crude oil immediately and left in the tank for 48 h. After 48 h [25,26,27], specimens were sacrificed, and approximately 50 µL of peripheral blood were collected from the caudal vein to perform genotoxicity tests. Fish husbandry and experimental procedures were conducted to conform with the EU legislation for the protection of animals used for scientific purposes and according to article 1, paragraph 5: no sufferance was applied to the organisms during experimental procedures [28].

### 2.4. DNA Primary Damage

*D. labrax* erythrocytes were processed for the evaluation of DNA integrity by the comet assay. The comet assay was performed in its mild alkaline version (pH 12.1) in order to detect exclusively single strand breaks (SSB) due to the abundance of alkali labile sites in functionally highly condensed chromatin characteristic of fish erythrocytes [29]. Before performing the comet assay, cell viability was checked to avoid false positive results. The Trypan Blue exclusion method always highlighted a percentage of viable cells higher than 95%, allowing us to proceed. Erythrocytes embedded in 0.5% low melting agarose (LMA) were dropped onto pre-coated microscope slides (dipped in 1% normal melting agarose, NMA, and air-dried overnight). After at least 1 h in lysing solution (2.5 M NaCl, 10 mM Tris, 0.1 M EDTA, 1% Triton X-100 and 10% DMSO, pH 10), slides were placed on a horizontal electrophoresis chamber and erythrocytes’ DNA was allowed to unwind for 10 min. Afterwards, a 10 min electrophoresis run was performed at 25 V, 300 mA, pH 12.1. To neutralize the pH, at the end of the run the slides were washed three times, 5 min each, with 0.4 M Tris, then put in 100% cold methanol for 3 min and allowed to dry. After staining with ethidium bromide, samples were observed under fluorescence at 400× magnification. Two slides per fish were setup, 50 random nuclei per slide were scored and then the mean was calculated. The percentage of tail DNA (i.e., the percentage of DNA migrating out of the nucleus), a reliable comet assay parameter [30], was chosen to evaluate the extent of DNA damage. It was calculated by an image analyzer (Komet 5.0 Software, Kinetic Imaging Ltd Bromborough, Wirral, Merseyside, UK, 2005) connected to the fluorescent microscope (% tail DNA).

### 2.5. Statistical Analysis

Multifactor analysis of variance (MANOVA) was carried out by considering the treatments as independent variables. The multiple range test (MRT) was performed in order to detect differences (*p* < 0.05) among different treatment groups.

## 3. Results

### 3.1. n-TiO_2_ Powder Characterization

**Figure 1:**
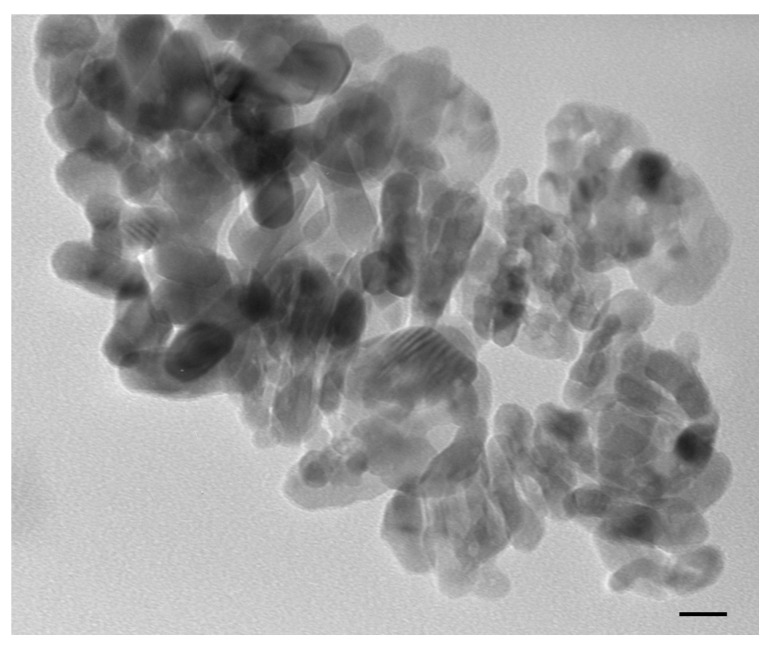
Transmission electron microscopy image showing aggregation pattern of n-TiO_2_ in water after sonication. Scale bar: 20 nm.

Transmission electron micrograph showed that after 1 h of sonication, n-TiO_2_ anatase particles coalesced in exposure media, forming variously sized aggregates (Figure 1).

### 3.2. In Vivo Exposure

**Figure 2:**
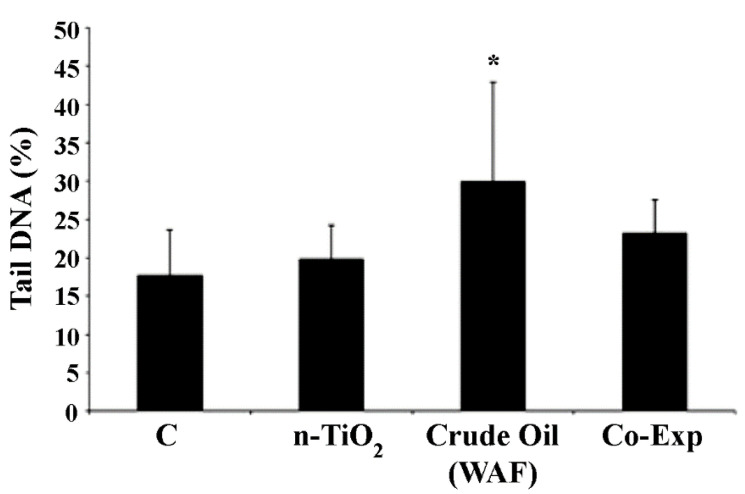
Levels of DNA damage in blood erythrocytes of *D. labrax* exposed to n-TiO_2_ and WAF crude oil, alone and in co-exposure (Co-Exp). Results are shown as mean ± SD. (*) indicates significant difference respect to the control group (C), * *p* < 0.05, (*n* = 5 for each experimental group).

The comet assay results showed a statistically significant increase (*p* < 0.05) of DNA damage in erythrocytes from juvenile specimens of European sea bass exposed to WAF of crude oil, and a lack of genotoxic effect exerted by n-TiO_2_ alone. Moreover, looking at the co-exposure, a significant recovery of induced DNA damage was observed (Figure 2).

## 4. Discussion

In the present preliminary study, selected pure anatase TiO_2_ nanoparticles were investigated to see if they exerted any genotoxic effect, and their capability to reduce WAF crude oil-induced genotoxicity was in vivo studied for the first time.

The water accommodated fraction (WAF) of crude oil is supposed to contain the highest concentration of dissolved petroleum hydrocarbons forecast from a spill [31,32]. In fact, the WAF represent a mixture of mono-aromatic (benzene, toluene, ethyl benzene and xylene), polycyclic aromatic hydrocarbons (PAHs), phenols and heterocyclic compounds, containing nitrogen and sulfur [33]. Therefore, WAF contains the most toxic substances in oil to aquatic species [34] and is potentially highly toxic to a wide spectrum of marine animals, confirming the classification from the International Agency for Research on Cancer that considered some petroleum-derived hydrocarbons carcinogenic and mutagenic contaminants [35].

Singer et al. [36] suggested the oil WAF be used for exposure media in toxicity experiments; in order to study oil contamination risk for aquatic organisms, and in the present in vivo study, sea bass (*Dicentrarchus labrax*) was selected as the model species. Sea bass are representative of estuarine species. They are typically bred for human consumption [37] and were demonstrated to be very sensitive in revealing genotoxic effects induced by polycyclic aromatic hydrocarbons (PAHs) and PAH-like compounds in peripheral erythrocytes [38]. The results presented report WAF crude oil able to exert a genotoxic effect in terms of DNA single strand breaks induction in erythrocytes of exposed sea bass.

Our results, although preliminary, confirm what was reported from exposure studies showing that petroleum hydrocarbons and their derivatives are harmful to aquatic animals, particularly fishes [39]. Moreover, such exposure may cause genotoxic, mutagenic and carcinogenic effects [40,41]. Genotoxic effects in erythrocytes and histopathological alterations in gills were observed in *C. parallelus*, and the authors attributed the cause to mono-aromatic and polycyclic aromatic hydrocarbons [3]. These compounds were also found to induce oxidative stress [42]. There is a relationship between oxidative stress and DNA damage expressed as SSB. The absence of n-TiO_2_ genotoxic effect was also demonstrated in our experimental set. In fact, specimens exposed to n-TiO_2_ alone showed levels of DNA SSB comparable with the control group specimens. The tendency of pure n-TiO_2_ anatase to form macro-sized agglomerate, as revealed from TEM primary characterization, indicates the inability of the particles to exert a direct genotoxic effect at the nanoscale level. This highlight is in line with what was reported by our previous in vitro study, which indicated n-TiO_2_ pure anatase as a cyto- and genotoxicity-free nanomaterial in respect to other n-TiO_2_ nanoparticles [14]. The results of our previous in vitro study guided us in choosing the nano-powder to be used for the present in vivo exposure research.

The debate on the toxicity of nano-titanium indicates the crystalline composition as a critical parameter. Mixtures of rutile and anatase are reported to affect antioxidant capacity and immune system, and to exert neurotoxicity in in vivo-exposed neotropical detritivorous fish, *Prochilodus lineatus* [43], genotoxic effects in treated *Mytilus galloprovincialis’* gill biopsies [14] and functional and molecular immune parameter alterations in in vivo-exposed marine bivalves [44].

In vivo fish exposure alterations in antioxidant activity in *C. gariepinus* treated with titanium dioxide pure anatase [45] have been reported, and such findings resulted differently from what was observed with other biological end-points in marine bivalve in vitro models [14]. This highlights that results depend on the experimental exposure (in vivo/in vitro), type of cell or tissue and biomarker analyzed, [46] and underlines the importance of a multi-level approach and that in-depth studies should be conducted both in vitro and in vivo for promoting nano-TiO_2_ safe application for marine organisms.

Fish species have been widely used as an indicator of pollution and are chosen as sentinel species to study aquatic toxicity since they strongly respond to stress condition effects [47]. The present pilot experiment confirmed the sensitivity of the marine fish used as a sensitive and useful model species in the research field of emerging pollutant genotoxic potential investigation and water decontamination pollutant efficacy technique assessment.

Another interesting point highlighted by this pilot study is that n-TiO_2_ pure anatase did not exert genotoxicity at dose and time tested. Moreover, it was also shown to be capable of handling WAF crude oil genotoxic effects when using the present vertebrate experimental model and in the present experimental condition, representing an important novelty for marine water nanoremediation within a laboratory setting. In fact, specimens exposed to the co-exposure (n-TiO_2_ and WAF crude oil) treatment showed a reduction of DNA damage in comparison with those exposed to WAF crude oil alone, and the analysis of other biochemical markers (data not shown) suggests the need of an in-depth investigation on the suitability of nTiO_2_ pure anatase in the field of marine water contamination following oil spills. Recently, the semiconductor titanium dioxide was suggested for use in the presence of UV light in photocatalytic disintegration of non-polar pollutants, such as petrol-derived hydrocarbons in water treatment [48].

The degrading capacity of titanium dioxide, in presence of sunlight irradiation, appears to be linked with the formation of highly reactive oxygen species that, as suggested by Pedanekar et al. [49], can disintegrate organic molecules (e.g., diesel fuel components) to their mineral constituent. Although the aim of this short communication is not to investigate the mechanism of action of n-TiO_2_, the previous consideration must also be considered for NPs genotoxic potential evaluation. Reactive oxygen species can induce DNA damage, although not under the experimental conditions discussed. In fact, in other experimental models, such as human epidermal cells, TiO_2_ nanoparticles were demonstrated to induce reactive oxygen species and oxidative stress leading to both oxidative DNA and chromosomal damage [50]. In regard to the interaction between TiO_2_ nanoparticles and classical contaminants, synergistic effects were observed in marine bivalves since significantly higher uptake of dioxin (2,3,7,8-TCDD) was detected in the co-exposed organisms [46]. Meanwhile, any interference was observed in co-exposed sea bass since nano-TiO_2_ did not interfere with dioxin detoxification and the bioconcentration capacity in the fish [19]. On the other hand, looking at the same nano-powders in the presence of cadmium, a Trojan horse effect was not observed. In fact, in cadmium co-exposed marine bivalves and sea bass, titanium dioxide nanoparticles seem to modulate the toxicological response to cadmium [19], and the co-exposure prevents the chromosomal damage and leads to a partial recovery of the genome template stability [29]. Looking at this complex background, future investigation with new cellular endpoints, longer exposure times (sub-chronic and chronic exposure) are of interest and should be performed. Moreover, correlation between biological responses and illumination conditions should be implemented in future nano-eco-toxicological research [51] because of the photocatalytic properties of nano-TiO_2_ that may result in alterations of their behavior in the environment, causing effects that have not yet been fully elucidated for the biota. The present preliminary results, although limited to genotoxicity evaluation in a fish species after an acute exposure (48 h), appear promising for potential applications of nano-TiO_2_ in environmental remediation of WAF crude oil contaminated sea water. Even so, the conflicting results reported in literature on n-TiO_2_ toxicity should pose caution in the applicative perspectives of these results.

In conclusion, in the present experimental conditions, pure anatase n-TiO_2_ was found to be a potential candidate to face marine pollutants, being harmless in terms of DNA primary damage induction and able to reduce the genotoxic effects induced by organic contaminants, such as crude oil WAF. *D. labrax* was also confirmed to be a sensitive marine vertebrate model to test nanoparticle genotoxic potential at the laboratory scale.

## Data Availability

Data are contained within the article.
